# Identification of a New Phosphorylated Host Interactor of the Epstein–Barr Virus (EBV) Kinase BGLF4 Suggests Key Points for EBV-Specific Antiviral Drug Targeting

**DOI:** 10.3390/ijms27062627

**Published:** 2026-03-13

**Authors:** Melanie Kögler, Christina Wangen, Alena Hammerschmitt, Debora Obergfäll, Friedrich Hahn, Manfred Marschall

**Affiliations:** Harald zur Hausen Institute of Virology, Friedrich-Alexander-Universität Erlangen-Nürnberg (FAU), Schlossgarten 4, 91054 Erlangen, Germany; melanie.koegler@fau.de (M.K.); christina.wangen@uk-erlangen.de (C.W.); alena.hammerschmitt@fau.de (A.H.); debora.obergfaell@fau.de (D.O.);

**Keywords:** Epstein–Barr virus (EBV), viral kinase BGLF4, host pyruvate dehydrogenase (PDH), mass spectrometry-based identification of BGLF4–PDH interaction, PDH phosphorylation by in vitro BGLF4 activity, kinase inhibitors with antiviral potential, novel options of anti-EBV drug targeting

## Abstract

Epstein–Barr virus (EBV) is a human pathogenic and oncogenic herpesvirus, with worldwide importance, at times associated with serious to life-threatening symptoms, especially in immunocompromised hosts. The available preventive options against EBV disease are limited to medically elaborate and cost-intensive measures of cell-based immunotherapy. The development of novel options of anti-EBV drug targeting is currently a matter of intense international efforts. A putative target of the antiviral therapy approach is the EBV-encoded protein kinase BGLF4, which fulfills a multifaceted role in productive viral replication. So far, viral BGLF4 interactor proteins and phosphorylated substrates have occasionally been reported, but in particular cellular interactors await further characterization concerning both, their relevance for BGLF4 functionality and their accessibility to antiviral drugs. In this study, we have analyzed host cell–BGLF4 interaction, BGLF4 kinase properties, and BGLF4-directed small molecules. The main results are as follows: **(i)** a mass spectrometry-based interactomic study was performed with EBV-producing Akata-BX1 cells, thereby identifying the human pyruvate dehydrogenase (PDH) as a relevant BGLF4 interactor; **(ii)** BGLF4–PDH interaction was confirmed by protein coimmunoprecipitation, subcellular cofractionation, and confocal imaging; **(iii)** the BGLF4-mediated phosphorylation of PDH was demonstrated by an in vitro kinase assay (IVKA); **(iv)** a reduction in PDH phosphorylation was shown for selected kinase inhibitors, which also exerted BGLF4-directed inhibitory potential in a quantitative qSox-IVKA, and **(v)** these hit compounds showed anti-EBV activity in lytically induced P3HR-1 cells using qPCR measurement, as well as PDH-inhibitory activity using standardized PDH assays. These data lead to an improved understanding of EBV–host interaction that may open novel anti-EBV preventive opportunities. Combined, the findings point to PDH as a new cellular interactor of the EBV kinase BGLF4. Also, notably, the data on pharmacological intervention with kinase activity or substrate phosphorylation may possibly provide as yet untapped options of antiviral drug targeting.

## 1. Introduction

Epstein–Barr virus (EBV) is a world-wide distributed γ-herpesvirus that is present in ≥95% of the human adult population and carries the ability to immortalize human cells. A global burden of approximately 164,000 deaths per year is linked to EBV infection. EBV-associated cancers are mostly induced by latent infection, and these include Burkitt’s lymphoma (BL), Hodgkin’s lymphoma (HL), EBV-positive diffuse large B cell lymphoma (DL-BCL), posttransplant lymphoproliferative disease (PTLD), as well as gastric and nasopharyngeal carcinoma (NPC) [1]. Viral tumor-specific pathogenesis is largely dependent on latently expressed viral oncoproteins (such as LMP1 and members of the EBNA group), but proteins expressed from the lytic program may additionally play accessory roles [2]. Moreover, EBV-associated disease, in which the viral lytic replication causes pathogenic dysregulation, likewise possesses clinical importance, i.e., chronically active infection (CAEBV), infectious mononucleosis (IM), post-transplant EBV disease (PTLD), or AIDS-related oral hairy leukoplakia (OHL) [3,4]. Thus, EBV-associated disease is partly linked to latent infection, while other symptomatic courses of EBV infection can arise from lytic replication. In all these cases, the EBV–host interaction, as based on regulatory protein complexes, is fundamental for dysregulatory cellular developments. In addition, during the last few years, the long-standing assumption of EBV association with the clinical development of multiple sclerosis (MS) could have been substantiated by several comprehensive studies [5,6,7]. Of note, the direct targeting of viral oncoproteins by antiviral drugs has not been achieved yet, but those diseases linked to EBV production and high viral load (e.g., CAEBV, IM, OHL, MS) should be accessible to lytic-phase inhibitors. Importantly, it should be emphasized that during the post-transplant situation, EBV can be massively reactivated, so that therapeutic prevention may suppress disease development (e.g., PTLD and long-term consequences).

BGLF4 is an EBV-encoded protein kinase, which shares structure-predicted and functionally validated similarities with human cyclin-dependent kinases (CDKs) and the cytomegalovirus vCDK/pUL97 [8,9,10,11]. These protein kinases interact with a number of cellular and viral proteins, to enable an efficient viral replication through the phosphorylation-mediated regulation of their substrates. Similar to the homologous protein kinases of other herpesviruses, BGLF4 plays an important role in the regulation of EBV nucleocytoplasmic capsid egress [12]. Additional functions have been assigned to BGLF4 in the suppression of the innate host immune response [13] and the nuclear import regulation of viral proteins [14]. BGLF4 is primarily produced during the early phase of viral lytic replication, in the nucleus of EBV-positive cells, but to lower quantities BGLF4 can also be detected in the cytoplasm, during the late phase of infection [15]. A pharmacological inhibition of BGLF4 is accompanied by reduced levels of virion production and release [16]. BGLF4 represents a viral tegument protein that is packaged into virions, and may thus fulfill functions already very early during the viral replication. It has been described that BGLF4 can interact with the viral lytic switch transactivator BZLF1 [17]. This may possibly happen already at immediate early time points of infection, or during viral reactivation. Thereby, BGLF4 has the potency to stimulate BZLF1 activity, even through site-specific phosphorylation [17,18]. Moreover, the BGLF4 kinase phosphorylates a number of further viral and cellular substrates. These may include the BGLF4-interacting proteins phosphatidylinositol 3-kinase-related kinase ATM [19], small ubiquitin-like modifier SUMO [20,21], minichromosome maintenance protein MCM4 [22], and possibly condensin and topoisomerase II [23]. In all cases of host—protein interaction, the functional relevance of BGLF4-mediated phosphorylation still awaits more detailed analysis to fully understand the regulatory networks. Moreover, the entire spectrum of host and virus protein interactions may illustrate the postulated multifaceted relevance of BGLF4 in EBV replication, and possibly even EBV-induced tumorigenesis, thus strengthening the approach of developing BGLF4-directed drugs.

A clearer role of BGLF4–host interaction was demonstrated for the process of viral nuclear egress. This gain in knowledge was mostly driven by the comparison of EBV BGLF4 with homologous kinases, in particular with human cytomegalovirus (HCMV) pUL97 [11,24,25]. During viral nuclear egress, BGLF4 and functionally related herpesviral kinases are responsible for the local disassembly of the nuclear envelope by site-specific phosphorylation of nuclear lamins A/C [12,24,26]. In this context, it is additionally interesting that BGLF4 associates with the nuclear envelope by binding to nucleoporins of the nuclear pore complex [27]. Thus, the potential direct or indirect interactions of BGLF4 with additional nuclear host proteins, like lamin A/C, emerin, or cyclins remains unclarified so far. Nevertheless, a network of BGLF4 binding partners appears obvious, and these may even reach beyond the host cell nucleus. In this study, we identified and characterized a new host interactor of the EBV protein kinase BGLF4, which has initially been characterized as a mitochondrial protein. We have analyzed the patterns of phosphorylation, the inhibitory activities of kinase inhibitors, as well as the anti-EBV potential of respective small molecules. In essence, the aspects of BGLF4-specific regulatory virus–host interaction and options of antiviral drug targeting (especially clinical situations, in which lytic EBV replication leads to pathogenesis, i.e., CAEBV, OHL, or PTLD) are discussed.

## 2. Results

### 2.1. Identification of a New Host Interactor of the EBV Protein Kinase BGLF4 by Applying a Mass Spectrometry-Based Interactomic Approach

Recently, we used mass spectrometry-based interactomics to identify host cell interactors of herpesviral protein kinases, especially the HCMV kinase pUL97 [28,29]. In this context, we and others raised experimental evidence for a number of very interesting pUL97-associated cellular factors, such as p32/gC1qR (bridging pUL97 to the viral nuclear egress complex), human cyclins (B1, T1, H), retinoblastoma protein (Rb), and interferon-induced innate or intrinsic immune factors (IFI16, SAMHD1) [29,30,31,32,33,34]. These putative pUL97-specific regulators were then functionally validated by activity-based approaches (reviewed in [10]). Unexpectedly, we also found the mitochondrial enzyme pyruvate dehydrogenase (PDH) among the screening hits for HCMV pUL97 [29]. Upon closer inspection, the mass spectrometry data revealed five subunits of the pyruvate dehydrogenase (PDH) complex to be among the most prominent hits [29]. The PDH subunits PDHB, DLST, DLAT, PDHX and DLD showed relatively high spectral count scores (SSC; 37, 42, 28, 26 and 21 respectively), and were absent from the control samples. These interactor SSC values, albeit not further characterized at this point, were similar to those of pUL97 itself (SSC 34.1). It appeared speculative whether the rather remarkably high abundance of PDH likely originated from the highly oligomeric nature of the PDH complex, containing 12–60 copies of its individual constituents. Although, we have not been able to verify the PDH interaction with the HCMV kinase pUL97 on a functional level, it did not escape our attention that PDH plays an interesting role in EBV-positive malignant B cell lymphoma [35]. In order to specifically address the putative association of PDH with the EBV kinase BGLF4, and to possibly identify further host interactors, we performed BGLF4-specific interactomic analyses. Here, we utilized EBV-positive Akata-BX1 cells [36], which were stimulated for lytic viral gene expression by the treatment with anti-human IgG. A set of samples was prepared by producing total cell lysates from 50 mL of Akata-BX1 culture each. Then, these lysates were subjected to standard CoIP as a sample quality control, based on protein G-coupled dynabeads, using a monoclonal antibody against BGLF4, or beads without antibody as a negative control (dynabeads control), respectively. Based on these samples, two independent replicates of analyses were used for the mass spectrometry-based approach (Figure S1). In a combined depiction of data, the weighted spectral count scores (WSC) were evaluated from the two replicates (Table 1; WSC ≥ 10 mAb-BGLF4.01 of Analyses 1 and 2), and were presented as the quotient relative to the negative control (WSC beads control). As an important result, the human pyruvate dehydrogenase (PDH) was indeed identified at highest WSCs in both experimental replicates (Table 1; PDH-specific WSC scores marked by blue frame). Hereby, WSC ≥ 10 served as the cut-off criteria in at least one of the replicates. Taking into account that the role of the host PDH for metabolic control in EBV-infected cells has been reported before [37,38], this is the first evidence for direct PDH interaction with an EBV protein. These data strongly suggest BGLF4–PDH complex formation in lytically stimulated EBV-producing Akata-BX1 cells. It should be mentioned that, in addition to the empty-beads negative control, two further specificity controls were used. On the one hand, a non-BGLF4-reactive control antibody (LSBIO LS-C331195) was used for assessing putative background reactivity in the Akata-BX1 lysate, but did not show relevant WSC signals for PDH (Figure S1). On the other hand, EBV-negative cells (J-Jhan), and other test samples, were used as a background control for mAb-BGLF4.01 in parallel. Due to the fact, however, that the latter background level in EBV-negative material was found relatively high, three different approaches of confirmation assays were performed as described by the procedures in the following section.

### 2.2. Confirmation of the Interactive Complex of BGLF4–PDH by Protein Coimmunoprecipitation, Subcellular Cofractionation, and Confocal Imaging

**Figure 1:**
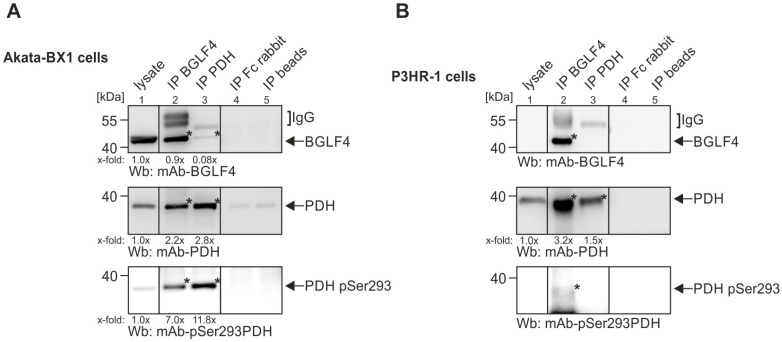
Confirmation of BGLF4–PDH interaction, as achieved by coimmunoprecipitation (CoIP). IgG-induced Akata-BX1 cells (**A**) or TPA-induced P3HR-1 cells (**B**) were harvested at 4 d post-induction and used for CoIP, applying the indicated IP antibodies. Note that corresponding CoIP bands were detected for both, IP BGLF4 and IP PDH, at expected molecular size ranges of approx. 42 kDa (BGLF4) and 38 kDa (PDH), respectively, although the results indicate an asymmetric CoIP detectability of the BGLF4–PDH interaction. A densitometric quantitation of the x-fold IP/CoIP band enrichment over lysate controls has been performed (mean values of determination in technical densitometric triplicates are given). The respective bands on the expected molecular sizes are indicated with asterisk *.

In the next step, we aimed to verify the mass spectrometry-detected complex of BGLF4–PDH by additional independent experimental approaches. First, we used direct and reverse coimmunoprecipitation analyses (CoIP). Therefore, total lysates were prepared from IgG-induced Akata-BX1 cells to perform a CoIP with BGLF4- or PDH-specific antibodies, respectively (Figure 1). Western blot (Wb) analyses using total cell lysates confirmed the expression of BGLF4 and PDH (lane 1). Applying either an IP monoclonal antibody against BGLF4 (lane 2) or against PDH (lane 3), positive results for CoIP were obtained (Figure 1, indicated on the right). Moreover, an antibody recognizing the phosphorylation-inactivated form of PDH (pSer293; [37]) likewise confirmed the interaction of BGLF4 and phosphorylated PDH (Figure 1, lower panel). Performing an analogous approach with the EBV-positive P3HR-1 cells, the PDH and its phosphorylated form coimmunoprecipitated with BGLF4 (Figure 1B). The specificity of the CoIP approaches was confirmed using a nonreactive Fc fragment (lane 4) or empty beads (lane 5). A densitometric quantitation, of the enrichment of bands in the BGLF4–PDH CoIP (x-fold enriched over lysate controls in lane 1), has additionally been performed (Figure 1A,B). It should be mentioned that the CoIP results show an asymmetric mode of detectable BGLF4–PDH interaction, with slight quantitative differences between signals in Akata-BX1 and P3HR-1 cells. The reasons may be given by CoIP effectiveness of antibodies or individual levels of protein expression. To this end, a quantitative indication has been provided by partial densitometric evaluation as indicated. Thus, the results were consistent with our mass spectrometry-based identification of PDH as a cellular interactor of EBV BGLF4.

**Figure 2:**
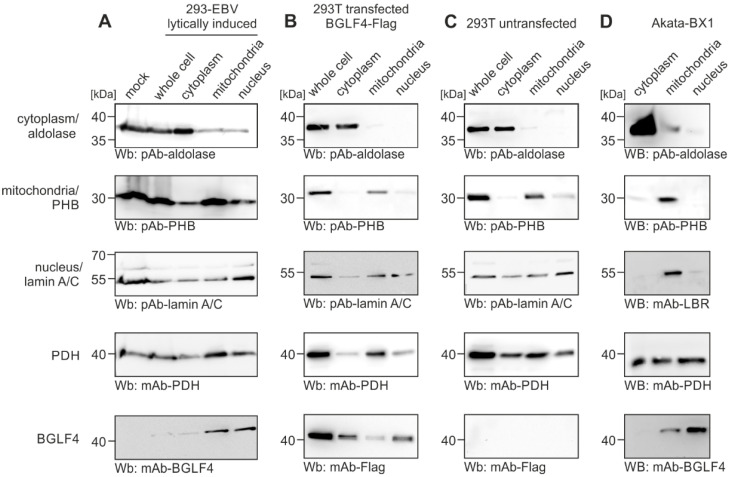
Cellular cofractionation experiment for addressing the subcellular colocation of viral BGLF4 and cellular PDH. Four different cellular settings were applied as follows: (**A**) 293-EBV cells, in which the recombinant EBV genome was lytically induced by the transfection of expression plasmids; (**B**) 293T cells transiently transfected with pcDNA-BGLF4-Flag; (**C**) 293T cells as an untransfected control; (**D**) Akata-BX1 cells (untreated). The expected proteins were enriched in their corresponding subcellular fractions with known molecular masses, i.e., aldolase (approx. 36 kDa), prohibitin/PHB (approx. 30 kDa), nuclear lamin A/C (approx. 53 kDa), nuclear lamin B receptor/LBR (approx. 55 kDa), pyruvate dehydrogenase/PDH (approx. 40 kDa), BGLF4 (approx. 42 kDa). The specific Wb staining of proteins was achieved by the use of antibodies as indicated beneath each panel.

In a second confirmatory approach, we performed cofractionation experiments with various cell settings: 293-EBV cells carrying a lytically induced EBV BACmid (Figure 2A), 293T cells transfected with BGLF4-Flag (Figure 2B), 293T cells untransfected/EBV-negative (Figure 2C), and EBV-positive Akata-BX1 cells (Figure 2D). This experiment aimed to detect postulated BGLF4–PDH interaction complexes within cellular fractions. To this end, cells were partially lysed and specifically processed to obtain samples enriched for cytoplasmic, mitochondrial and nuclear proteins (Figure 2, see top labeling of panels). The fractionation controls were represented by aldolase (cytoplasmic marker; upper panels), prohibitin (PHB, mitochondrial marker; second panels), and nuclear lamin A/C or nuclear lamin B receptor (nuclear marker; third panels). By the use of these markers, the separation could be monitored. This indicated a reliability of the cell fractions within the limits of the method. As a result, the endogenous PDH (fourth row of panels) was predominantly detected in the mitochondrial fraction, as detectable for most of the cell type preparations. In addition, this mitochondrial host protein also appeared, at lower quantities, in the nuclear and cytoplasmic samples of this cofractionation setting. Notably, the viral kinase BGLF4 (as expressed in A, B, and D) showed a cofractionation signal together with PDH in the nuclear and mitochondrial samples of EBV-positive cells (bottom row of panels). Interestingly, the patterns of BGLF4-transfected and untransfected 293T cells appeared slightly distinct (and were consistent with additional CoIP results using transfected 293T cells). Combined, although the discriminatory precision of this approach is generally considered as limited, the finding is consistent with a direct BGLF4–PDH interaction in EBV-positive cells.

**Figure 3:**
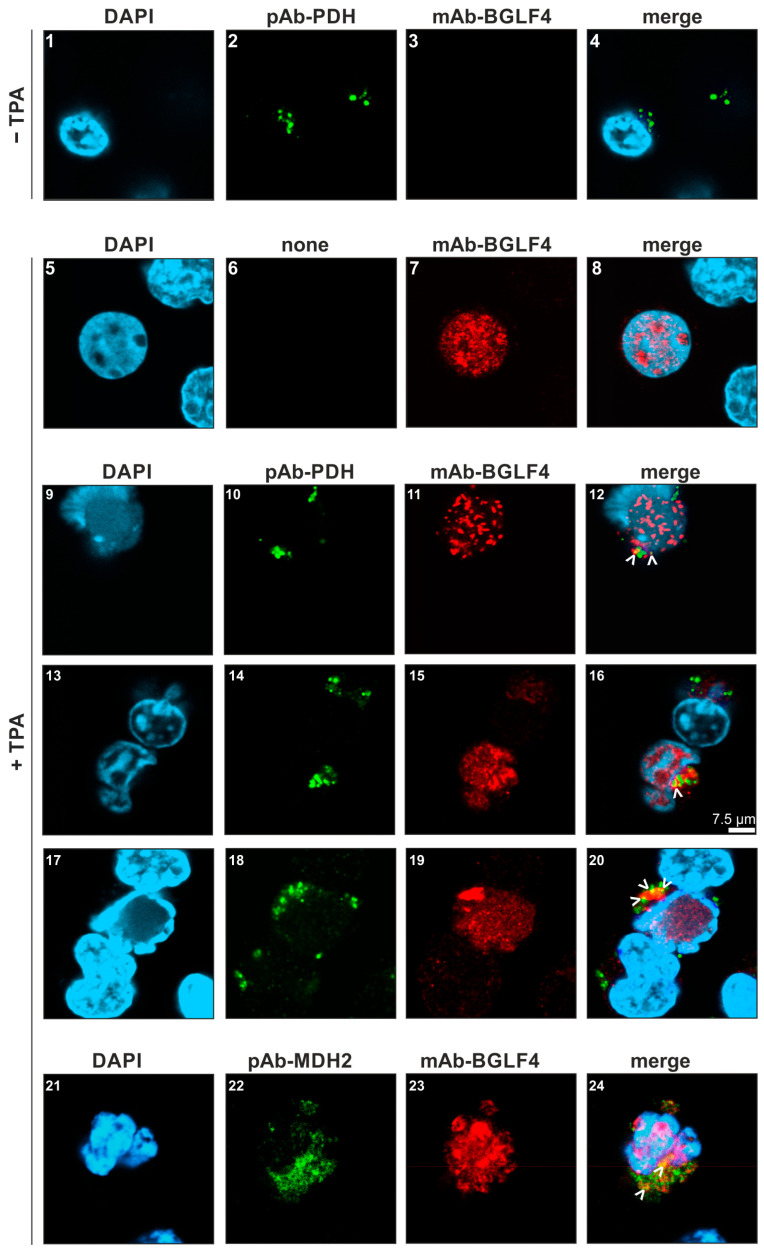
Confocal imaging of BGLF4 and PDH colocalization. P3HR-1 cells were either stimulated with TPA (80 ng/mL, 4 d; images (**5**–**20**)) or remained unstimulated (images (**1**–**4**)). Cells were seeded onto coverslips (collagen-coated 0.05 mg/mL) in serum-free medium and were processed by indirect immunofluorescence staining using the antibodies directed against BGLF4 and PDH as indicated (MDH2 was additionally stained as a mitochondrial marker, images (**21**–**24**)). BGLF4 was detected using Alexa Fluor 555 (red), while PDH was stained with Alexa Fluor 488 (green). Note that BGLF4 localized predominantly to the nucleus (DAPI staining), whereas PDH was mainly distributed at non-nuclear locations. Signals of extranuclear BGLF4–PDH colocalization were indicated by white arrows; scale bar 7.5 µm.

Then, to address a potential intracellular BGLF4–PDH colocalization, we performed indirect immunofluorescence costaining and confocal imaging (Figure 3). By analyzing lytically induced TPA-treated P3HR-1 cells, viral BGLF4 was mostly detected in the nuclei (Figure 3, images 7, 11, 15, 19). The cellular PDH was predominantly localized to cytoplasmic punctae, potentially representing mitochondria (images 2, 10, 14, 18). Interestingly, certain distinct speckles of BGLF4–PDH colocalization could be visualized (see merge panels, images 12, 16, 20, white arrows). These colocalization signals did not appear in the nuclei of BGLF4-positive cells but were mostly juxtaposed to the respective nuclear areas. The approach cannot exclude the existence of additional nuclear colocalization, but the relatively low abundance of PDH signals may point to limited sensitivity of the available antibodies in the confocal imaging method. The association of BGLF4 with mitochondria was further supported by a costaining with the mitochondrial marker protein MDH2 (images 22, 23, 24; for complete set of MDH2-specific images, see Figure S2). It is a highly interesting, and rather unexpected, finding to reveal BGLF4–PDH colocalization mainly in cytoplasmic speckles obviously representing mitochondria. PDH is a typical mitochondrial protein, whereas BGLF4 is primarily localized to the nucleus. This may indicate that the interaction of the two proteins mediates, at least in part, the translocation of a PDH-associated fraction of BGLF4 to mitochondrial speckles. The consequences of this localization phenotype are still hypothetical but may either point to the regulatory impact of BGLF4 onto PDH in mitochondria, or, alternatively, may suggest a PDH-mediated deregulation of BGLF4. In essence, as supported by CoIP and cofractionation analyses, this confocal imaging approach provided further experimental evidence for BGLF4–PDH interactive complexes in EBV-positive cells.

### 2.3. Demonstration of the BGLF4-Mediated Phosphorylation of PDH by In Vitro Kinase Assay

**Figure 4:**
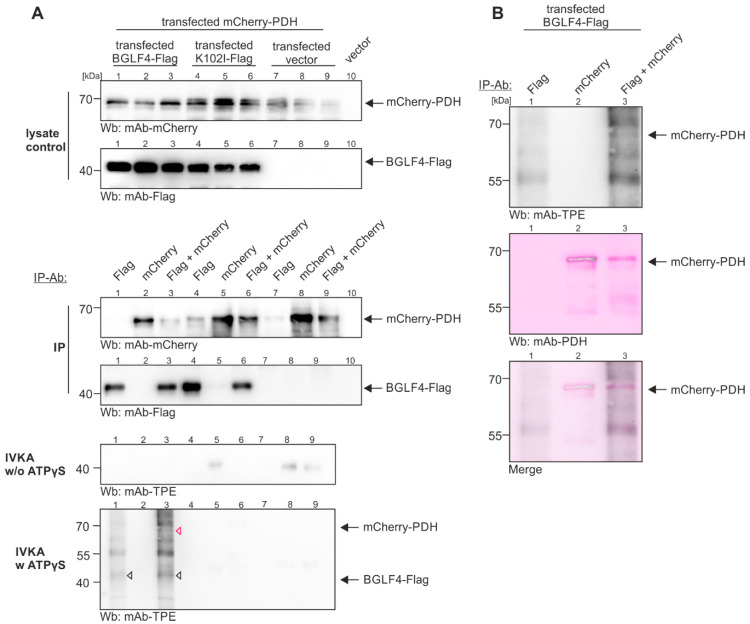
In vitro phosphorylation of PDH by BGLF4 using proteins from plasmid-cotransfected 293T cells. Cells were transiently transfected with expression plasmids for BGLF4 in its active form (BGLF4-Flag), a catalytically inactive mutant of BGLF4 (K102I-Flag), and/or an autofluorescent mCherry–PDH fusion protein (mCherry-PDH), as indicated. At 2 d post-transfection, (**A**) input lysate controls were taken (50 µL), before the main lysate volumes (450 µL) were subjected to immunoprecipitation using the antibodies indicated on top of the panels (IP-Ab). Based on the IP samples, an in vitro kinase assay (IVKA) was performed. To this end, the enriched proteins were subjected to the IVKA reaction with (w) or without (w/o) ATPγS (the latter used as a background control). Note, that BGLF4 autophosphorylation was observed at approx. 40 kDa (black triangle), in addition to the phosphorylation of mCherry-PDH at approx. 68 kDa (red triangle). (**B**) The IVKA blots (lanes 1–3) were subsequently restained with a PDH-specific antibody (pink color, see inset panels) to confirm the PDH specificity of BGLF4-mediated phosphorylation band. One representative experiment of two biological replicates is shown.

In the next step, we examined whether the experimental data supporting BGLF4–PDH interaction can be substantiated by demonstrating a BGLF4-mediated phosphorylation of PDH. Here, we used an established in vitro kinase assay (IVKA) under conditions described elsewhere [39]. To this end, BGLF4-Flag (catalytically active version, as compared to the inactive kinase dead-mutant K102I) and mCherry-PDH were expressed by transient transfection of 293T cells (Figure 4). The expression levels were monitored by Western blot (Wb) analysis using total cell lysates (Figure 4A, upper panels). The question of whether mCherry-PDH was subject to BGLF4-Flag-mediated phosphorylation was addressed by using tag-specific antibodies and antibody combinations for immunoprecipitation (IP-Ab). In the IVKA setting with ATPγS addition, but not in the absence of ATPγS (Figure 4, lower two panels), the BGLF4-Flag autophosphorylation (open white triangles) and the mCherry-PDH substrate phosphorylation (red triangle) were detectable. Control samples with the inactive mutant K102I, and vector controls, did not show the respective signals. The identity of the phosphorylation signal was subsequently verified as PDH by restaining of the respective band with the specific antibody (Figure 4B). Note, that lanes 5, 8, and 9 of the IVKA negative control panel w/o ATPγS show some background signals, which, however, do not interfere with the positive phosphorylation signals of mCherry-PDH/BGLF4-Flag in lanes 3 and 1 (in addition, this background has been addressed and reduced in a second experimental replicate shown in Figure S3). To validate the assay specificity, negative controls have been used in parallel experiments, such as related herpesviral protein kinases (pUL97), unphosphorylated control proteins, and no-protein controls. Thus, the results strongly support our notion that the BGLF4–PDH interaction may represent a virus-specific cellular substrate phosphorylation.

### 2.4. Anti-EBV Activity of Selected Kinase Inhibitors, Determined in a qPCR-Based Antiviral Assay

**Figure 5:**
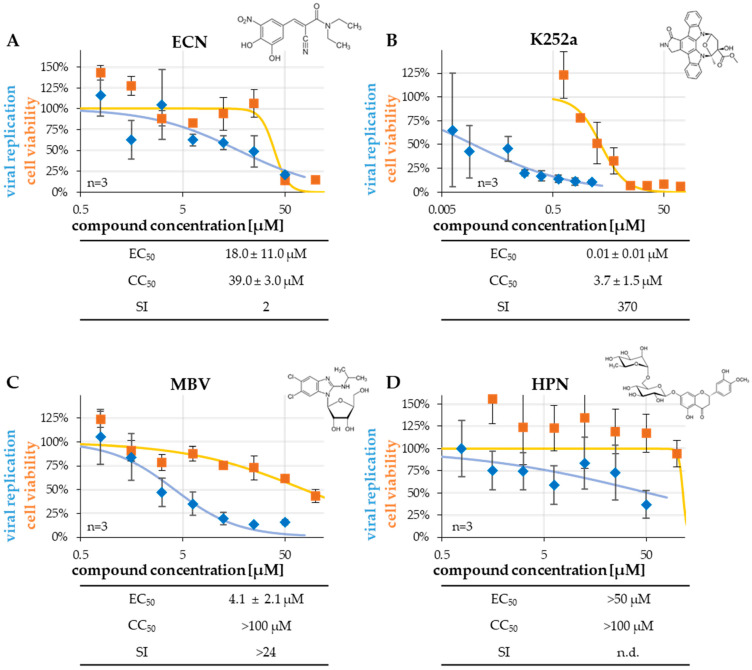
The clinically relevant small molecules entacapone (ECN) (**A**), staurosporine analog (K252a) (**B**), maribavir (MBV, comparative antiviral drug) (**C**) and hesperidin (HPN) (**D**) were analyzed for their antiviral activity. In the P3HR-1-based qPCR assay, cells were stimulated with TPA (80 ng/mL, 10 d), and the inhibitory compounds were administered immediately after induction in a two-fold dilution series, before qPCR was conducted at 10 d post-stimulation (blue curve). Additionally, cell viability of unstimulated cells was assessed using a resazurin-based assay (orange curve). Data are presented as mean ± SD of triplicate measurements derived from three independent replicates.

In order to seek BGLF4-inhibitory small molecules, the anti-EBV activity of selected kinase inhibitors was addressed using a recently established antiviral assay system [40] (Figure 5). The inhibitors were either already known/supposed to be directed to the viral kinase BGLF4, or to host kinases, or possibly to both: the clinical drug entacapone (ECN; Figure S4) and the bioflavanoid hesperidin (HPN; Marschall et al., unpublished data) have both recently shown interesting characteristics in our in vitro kinase assays performed on a selection of herpesviral and cellular CDK-like protein kinases. K252a is a compound that has been described as a potent inhibitor of EBV replication in cell culture-based analysis [41]. Also, maribavir (MBV), as an approved antiviral drug against HCMV infections, has been used in parallel (as a comparative antiviral compound). MBV is considered as a highly selective pharmacological inhibitor of herpesviral kinase activity (mainly HCMV pUL97, as strongly supported by our previous reports [42,43]. The experimental compounds ECN, HPN, and K252a most probably exert a broader selectivity panel including secondary targets. With this selection of partly clinically approved and partly experimentally analyzed drugs, we intended to quantitate, first, the overall anti-EBV potency in an EBV replication system, and subsequently the potential BGLF4-specific inhibitory activity. The EBV lytic replicative activity was measured in the EBV-positive immortalized B cell line P3HR-1, chemically induced by stimulation with 80 ng/mL of 12-O-tetradecanoylphorbol-13-acetate (TPA) for 10 d, to assess the EBV lytic productivity. For this purpose, P3HR-1 cells were seeded in 96-well plates, simultaneously induced with TPA, and treated with antiviral compounds for 10 d, before cell culture medium samples were subjected to the EBV-specific qPCR, as described before [36,40]. In this setting, a pronounced antiviral activity was observed for MBV and K252a with half-maximal effective concentrations of 50% (EC_50_ values of 4.1 ± 2.1 µM and 0.01 ± 0.01 µM, respectively), while ECN showed intermediate-level activity (18.0 ± 11.0 µM), and HPN was inactive (>50 µM; Figure 5). The data available so far suggest that K252a and ECN may represent BGLF4-directed anti-EBV compounds, whereas the reference drug MBV acts through a different mechanism, and HPN shows no anti-EBV activity. Moreover, cell viability was expressed as the lack of cytotoxicity in the range of concentrations relevant for antiviral activity (see CC_50_ values/half-maximal cytotoxic concentrations of 50%). The SI values (quotients CC_50_/EC_50_) underlined the noncytotoxic inhibitory potential of K252a, (restrictedly) ECN, and MBV in this system.

### 2.5. Inhibition of Viral BGLF4 In Vitro Phosphorylation Activity by Selected Kinase Inhibitors Using qSox-IVKA

**Figure 6:**
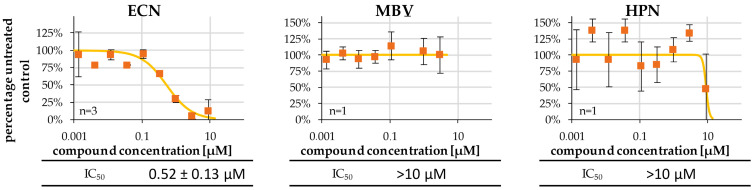
Putative kinase inhibitors were analyzed for their potential to inhibit the BGLF4 catalytic in vitro activity using the qSox-IVKA. 293T cells were seeded in 10-cm^2^ dishes and transfected with BGLF4-Flag plasmid. Two days post-transfection, cells were lysed and BGLF4-Flag was immunoprecipitated. Immunoprecipitates were resuspended in 50 µL enzyme buffer and subjected to qSox-IVKA. For each reaction, 45 µL of kinase reaction mix was added to Corning NBV 96-well half-area microplates along with 5 µL of the respective compounds diluted in a three-fold series. Kinase activity was monitored using a Victor multilabel reader at 30 s intervals over approx. 90 min. Kinase inhibitory activity was normalized to the DMSO control, and IC_50_ values were determined for each compound. IC_50_ values are presented as mean ± SD of triplicate measurements (one representative replicate is shown).

The anti-EBV activity of the putative kinase inhibitors ECN, MBV and HPN raised the question of whether this activity was mechanistically linked to BGLF4 inhibition. To this end, phosphorylation of a kinase-specific sensor peptide by BGLF4, immunoprecipitated from transiently transfected 293T cells, was quantitatively determined by qSox-IVKA [43,44] (Figure 6). Signal levels of the untreated, fully active kinase were set to 100%, which could then be compared to serial dilutions of three potential inhibitors. As a relevant result, ECN showed a marked BGLF4-inhibitory activity, with a half-maximal inhibitory concentration of 50% (IC_50_) of 0.52 ± 0.13 µM, while MBV and HPN were negative in this setting (>10 µM). This finding confirmed that the HCMV kinase pUL97 inhibitor MBV proved to be BGLF4-negative. The combined findings, obtained by the antiviral assay and the qSox-IVKA, illustrate the difference between those anti-EBV compounds being BGLF4-directed (ECN, K252a), in contrast to others acting in a BGLF4-independent manner (MBV). Thus, the currently available data suggest that the anti-EBV activity of the investigated compounds was either based on a BGLF4-directed mode of action, or on a BGLF4-independent, so far unresolved mechanism.

### 2.6. Inhibition of BGLF4-Specific PDH Substrate Phosphorylation by the Identified Compounds, as Potentially Linked with Changes in PDH Activity

**Figure 7:**
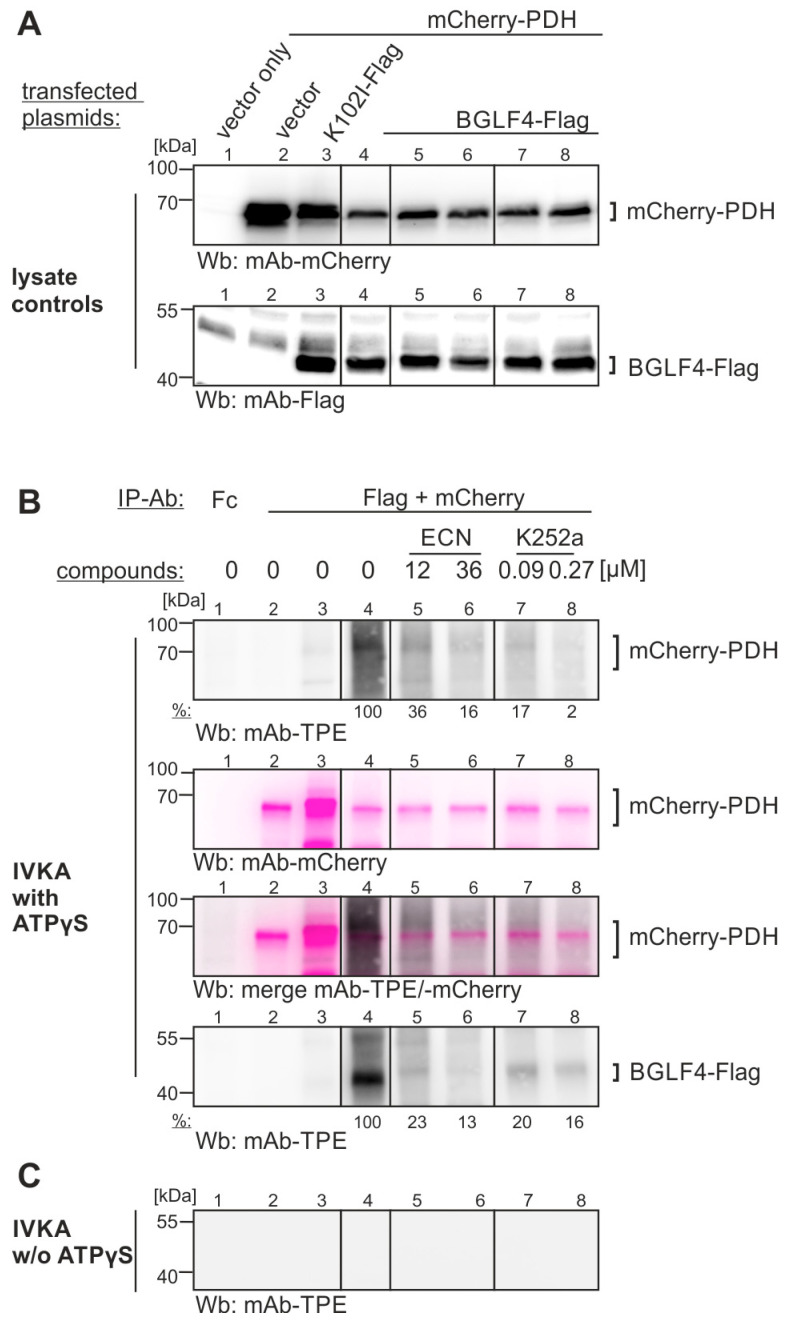
Specific inhibition of the BGLF4-mediated in vitro phosphorylation of PDH, by treatment with either of two identified anti-EBV compounds. (**A**) 293T cells were used for plasmid cotransfection as indicated (BGLF4-Flag active kinase; K102I-Flag catalytically inactive mutant; mCherry–PDH as putative substrate), before 2 d post-transfection, the cells were lysed and used for immunoprecipitation with the given antibodies (IP-Ab). Total cell lysate controls were taken before immunoprecipitation, to monitor the expression levels on Wb (for details, see Figure 4). The compounds ECN or K252a, or DMSO as a solvent control, were incubated in the culture media overnight before cell harvesting, and additionally during the IVKA reaction. (**B**) The immunoprecipitates were then subjected to the in vitro kinase assay (IVKA) under conditions (**B**) with ATPγS for phospho-detection or (**C**) without ATPγS for background control. The IVKA blots were subsequently restained with a PDH-specific antibody (pink color) to confirm the specificity of bands. Note, the mCherry-PDH-specific phosphorylation bands around 68 kDa, which were reduced in the case of both inhibitor treatments. The inhibitor concentrations were adjusted to their antiviral EC_50_ values (see Figure 5). One representative experiment of two biological replicates is shown. A densitometric quantitation of the percentage inhibitor effect regarding phosphorylation signals is indicated (% band reduction by inhibitor treatment versus control w/o inhibitor in lane 4; mean values of determination in technical densitometric triplicates are given).

In order to achieve a verification that the identified anti-EBV compounds may potentially inhibit BGLF4-specific substrate phosphorylation, a second setting of IVKA was performed. Here, transiently expressed kinase and substrate proteins were used as IP-based input materials. A focus was given to ECN and K252a, since both compounds exerted antiviral activity against EBV replication (see Figure 5) and qSox-IVKA-based inhibitory activity against BGLF4 (see Figure 6 or shown elsewhere), respectively [41,45]. In the present setting (Figure 7), mCherry-fused or Flag-tagged versions of mCherry-PDH (A1-N-10 vector) or BGLF4-Flag (pcDNA3.1 vector), respectively, were used for transient cotransfection of 293T cells, and appropriate expression levels were monitored by Wb analysis using tag-specific antibodies (Figure 7A). In the IVKA reaction (Figure 7B), the PDH-mediated phosphorylation by BGLF4 was detectable (lane 5, compared to the negative control lanes 1–4), and here, the incubation of antiviral compounds showed specific inhibitory effects. The PDH-directed substrate phosphorylation by BGLF4 was markedly reduced by both compounds, ECN (lanes 6 and 7) and K252a (lanes 8 and 9). The identity of mCherry-PDH-specific bands could be verified by Wb restaining (pink color). Likewise, the BGLF4 autophosphorylation was similarly reduced by ECN and K252a (lowest panel in B). No background signals were obtained in the absence of ATPγS as a specificity control (Figure 7C). These findings further illustrate the BGLF4-specific PDH substrate phosphorylation, and the inhibition by selected compounds. This suggests a novel target-accessible activity that may possibly be exploited by antiviral approaches.

**Figure 8:**
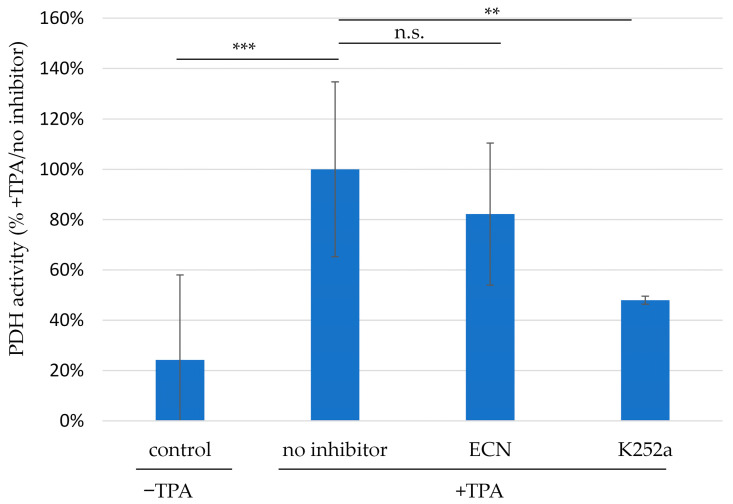
Measurement of PDH activity in TPA-stimulated EBV-positive Akata-BX1 cells, in the absence or presence of BGLF4-directed inhibitors (applying the PDH assay ab109902). Akata-BX1 cells were cultivated in media volumes of 6 mL per sample for 4 d. PDH activity was determined according to instructions of the manufacturer described in Section 4.9. From the performed biological replicates, one representative experiment is shown (see also Figure S5), and measurements were performed in technical triplicates. Mean values ± SD are shown, and Student’s *t*-test was applied for statistical evaluation. **, *p* > 0.01; ***, *p* > 0.001; n.s., not significant.

For addressing the question, whether PDH activity is modulated in lytically EBV-producing cells, the standard PDH assay system ab109902 was applied. For this purpose, Akata-BX1 cells were stimulated with TPA for 4 d, optionally under treatment with one of the identified BGLF4-directed inhibitors, ECN or K252a, or without inhibitor treatment as a control (Figure 8). The EBV-stimulated condition showed a marked increase in PDH activity versus control (bars 1–2). This finding is compatible with our assumption that EBV infection may have a regulatory effect on intracellular PDH activity. It should be noted that the current setting cannot rule out a change in PDH signals referring to varied PDH expression levels (possibly EBV-dependent) or an impact of TPA (possibly EBV-independent). Interestingly, however, both identified EBV inhibitors showed a partial blocking of the increase in PDH signal (ECN, bar 3, n.s.; K252a, bar 4, statistically significant). This argues for an EBV-specific effect, at least in part, directed to PDH activity that might involve the PDH-interacting BGLF4 kinase. Finally, a second, independent readout system was applied for this experimental setting (Figure S5). Data illustrated a similar trend, albeit lacking statistical significance at this early level of investigations. It has to be emphasized that the current experimental findings do not fully exclude alternative explanations, such as changes in PDH expression levels or effects associated with TPA-induced cellular responses. Nevertheless, one plausible interpretation of results points to a regulatory impact of BGLF4 on PDH. Combined, the data provide a first indication that conditions of lytic EBV production may be modulatory for PDH activity, and that BGLF4–PDH interaction may possibly correlate with this effect.

## 3. Discussion

The results of the present study reveal several points of evidence concerning properties of protein–protein interaction, substrate phosphorylation, intracellular localization, and drug sensitivity of the EBV kinase BGLF4. Notably, the results suggest a regulatory interplay between the two proteins BGLF4 and PDH, which both represent multifunctional regulators of the metabolism in EBV-infected cells. Although the data cannot yet prove a definite functional importance of the BGLF4–PDH interaction, for EBV replication or for cell-specific modulation, this is the first indication of a novel contact point in the complex field of EBV–host interaction. At this stage, however, the understanding of the biological significance of the BGLF4–PDH interaction requires additional investigations. A main impact of the current experimental findings lies in the recognition of a newly identified host factor with potential virus-supportive activity. A follow-up study of these regulatory issues, and a longer-term future investigation of the relevance of BGLF4 and PDH in models of EBV infection, at best in vitro and in vivo, may help to define these key points, especially in the direction of novel antiviral drug targeting.

The regulatory role of the EBV-encoded protein kinase BGLF4 has been illustrated by a number of previous reports [11,12,19,46,47,48,49]. BGLF4 represents, on the one hand, a virion tegument protein, and, on the other hand, is expressed with early protein kinetics within the viral productive replication cycle. It shares several functional properties with homologous kinases of γ- and β- herpesviruses, but to a much lesser extent with kinases of α-herpesviruses [10,28,46,50]. A number of viral BGLF4 interactors, and potential phospho-regulated substrate proteins, have previously been suggested [47,48,49,51,52,53]. In addition, the question of BGLF4–host interaction has been addressed [9,12,19,21,23,54]. So far, however, the functional role of these interactions, and the complexity of BGLF4 activities within the viral lytic replication cascade, have only incompletely been elucidated. In the present study, we identified a new host interactor of the EBV kinase BGLF4, the multifaceted cellular regulator PDH, which undergoes BGLF4-mediated phosphorylation, and may thereby exert regulatory importance for viral replication. The latter aspect requires more detailed investigations in the future, to possibly state a functional role in productive EBV replication. According to our present findings, the following statements could be made: (i) an interactomic study identified human PDH as the most relevant BGLF4-interacting protein, (ii) the BGLF4–PDH interaction was confirmed by various biochemical and cell-based methods, (iii) in vitro phosphorylation of PDH by BGLF4 was demonstrated (IVKA analysis); (iv) this PDH-directed phosphorylation activity was found reduced by kinase inhibitors; and (v) the hit kinase inhibitors proved anti-EBV activity. The latter results especially may help to define key points for EBV-specific antiviral drug targeting.

It is an outstanding feature of EBV pathobiology that long-term infection may induce two different types of viral pathogenicity. One type of EBV-associated disease is linked to viral latency, while the other type refers to productive replication. At present, the clinical options of anti-EBV treatment in cases of EBV-associated pathogenicity, e.g., under immunosuppressed conditions such as post-transplantation, are still strongly limited. Although EBV disease based on viral latency is not directly subject to replication-directed inhibitors, an indirect clinical benefit arising from anti-EBV drugs appears probable. It should be emphasized that even the establishment of viral latency (mostly in B lymphocytes) is based on a preceding, highly productive phase of lytic EBV infection (in tonsils, epithelia, gut, and other privileged sites). Against this background of knowledge, our current focus is the question of whether novel antivirals that inhibit EBV lytic replication can also provide a rate-limiting step of long-term clinical issues.

Trials with antiherpesviral nucleoside analogs have been largely disappointing [4], so novel options of inhibitor targeting are urgently needed [25,55]. Although novel direct antiviral targeting strategies have proven profoundly successful for other herpesviruses, e.g., applying inhibitors of viral terminase, kinase, or helicase [56], the efficacy of correlate drugs in EBV infection still await proof-of-concept. In this study, we are suggesting a novel host-derived target, with a previously suggested virus-supportive role in EBV infection, for putative anti-EBV targeting strategies. The pyruvate dehydrogenase (PDH) complex serves as a central checkpoint in cellular energy metabolism, linking glycolysis to the oxidative processes of the tricarboxylic acid cycle. Its catalytic function depends on the coordinated action of the E1, E2, and E3 subunits, which convert pyruvate into acetyl-CoA. PDH activity is primarily regulated by inhibitory phosphorylation, and reduced function of the complex, which shifts metabolism toward increased lactate production. This metabolic reprogramming reflects a broader decline in oxidative capacity and contributes to impaired energy homeostasis under stress or disease conditions [57,58,59,60]. Notably, lactate excretion is observed in cells freshly infected with EBV and is also a metabolic hallmark of NPC [38,61]. Since extracellular lactate exerts immunosuppressive functions, e.g., in tumor microenvironments, a BGLF4/PDH-directed therapy might be beneficial for the treatment of EBV-induced diseases [62]. This might be similarly relevant for other virus infections that show a modulation of PDH through viral proteins [4,63]. The specific regulatory function of PDH, within the multifaceted contact points of EBV–host cell interaction, has been relatively poorly investigated so far. Furthermore, the identification of PDH in a previous mass spectrometry dataset of pUL97-interacting proteins may suggest that HCMV [29], and potentially other human herpesviruses like EBV, could regulate PDH activity through phosphorylation by their respective viral kinases. On the basis of the present report, a detailed future analysis of the functional consequences of BGLF4–PDH interaction, may clarify these questions.

Recently, the scientific development and clinical approval of maribavir (MBV), as the first kinase inhibitor in the entire field of antiviral drugs, has set new benchmarks. Concerning the lack of in vitro inhibitory potential of MBV against EBV BGLF4, there has not been fully convincing evidence about an in vivo anti-EBV efficiency in previously published reports [41,64,65]. As a result of the present study, however, BGLF4 shows very interesting parameters of host—protein interaction, regulatory features of kinase activity, and sensitivity to inhibitory small molecules. Especially, the findings of the BGLF4-directed antiviral activity of ECN and K252a acts as promising support for our strategy. Hereby, the inhibition of EBV lytic replication and prevention of viral spread appears mostly relevant in acute, virus-driven proliferative conditions, such as IM, CAEBV, PTDL, or OHL. Thus, specifically in these situations, the strategy of utilizing BGLF4 as an anti-EBV drug target, appears attractive. Forthcoming molecular studies may clarify these attractive options, in terms of an improved understanding of virus-specific regulation, and translational antiviral developments against EBV infection.

## 4. Materials and Methods

### 4.1. Cells and Viruses

Akata-BX1 cells (human B lymphoblastoid cells carrying a GFP-expressing recombinant of Akata EBV strain; [66,67] were cultured in RPMI-1640 medium (Thermo Fisher Scientific, Waltham, MA, USA) supplemented with 10% FCS, 1xGlutaMAX^TM^, 10 g/mL gentamicin and 350 µg/mL geneticin. EBV Akata lytic gene expression was stimulated by the treatment with anti-human IgG (rabbit IgG anti-human (Fc)-unconjugated, 0.4 mg/mL for 3–7 d, Dianova, Hamburg, Germany). P3HR-1 cells were cultivated in RPMI-1640 medium with 10% FCS, 1xGlutaMAX^TM^ and 10 g/mL gentamicin. EBV P3HR-1 lytic gene expression was stimulated by the treatment with TPA (12-O-tetradecanoyl-phorbol-13-acetate, 40–80 ng/mL, 4–10 d, Selleck Chemicals LLC, Houston, TX, USA). The EBV BACmid-carrying 293 producer cells (gift from Christian Münz, Institute of Experimental Immunology, University of Zürich, Zürich, Switzerland) were cultured in RPMI-1640 medium supplemented with 10% FCS, 1xGlutaMAX^TM^, 100 µg/mL penicillin-streptomycin and 100 µg/mL hygromycin (InvivoGen, Toulouse, France). Lytic induction of virus production in 293-EBV cells was achieved by the transient transfection with the plasmids p509 (BZLF1) and p2670 (BALF4) [68]. HEK293T cells were cultivated in Dulbecco’s modified Eagle’s medium (DMEM) containing 10% FCS, 1xGlutaMAXTM and 10 g/mL gentamicin cells were cultivated at 5% CO_2_, 37 °C, and 80% humidity and were regularly checked for mycoplasma contamination using the Lonza™ Mycoalert™ kit (Thermo Fisher Scientific, Waltham, MA, USA).

### 4.2. Antiviral Compounds

Inhibitors used in this study were entacapone (ECN, biomol Cay14153-10), staurosporine analog (K252a, biomol, Cay11338-1), maribavir (MBV, a comparative antiviral compound, Shanghai PI Chemicals Ltd., Shanghai, China) and hesperidin (HPN, Selleckchem, S1529, Shanghai, China).

### 4.3. Sample Preparation for Mass Spectrometry Analysis and Quality Control

For mass spectrometry analysis Akata-BX1 cells were cultivated as described in Section 4.1. To induce lytic viral gene expression, the cells were stimulated with human anti-human IgG (rabbit IgG anti-human Fc, unconjugated, 0.4 mg/mL; Dianova, Hamburg, Germany). After 4 days, 50 mL of stimulated Akata-BX1 culture was harvested to perform a coimmunoprecipitation (CoIP) as described in Section 4.5. The antibodies used for immunoprecipitation were mAb-BGLF4 (mAb-BGLF4.01, Tihana Lenac, Rijeka, Croatia), a non-BGLF4-reactive control antibody (LSBIO LS-C331195), and empty Protein G-Dynabeads (Thermo Fisher Scientific, USA) as an additional negative control. Aliquots of the samples were prepared for quality control stainings, in the first step, and mass spectrometry analysis in the second step. To validate the quality of the immunoprecipitated proteins, samples were analyzed qualitatively and quantitatively. The samples were first analyzed to ensure successful IP of BGLF4 via standard Western blot (Wb) detection using antibodies as indicated. Subsequently, proteins were separated on 12.5% SDS-PAGE gels and stained with InstantBlue^TM^ (Expedeon, San Diego, CA, USA) for 1 h at room temperature under continuous gentle agitation. The gels were then subjected to standard silver staining.

### 4.4. Subcellular Cofractionation

Subcellular cofractionation was performed as described in [69] with some minor modifications. Instead of mechanically disrupting the cells, sonification was carried out. Fractions were analyzed using following antibodies: pAb-aldolase (CHEMICON International AB1809, Temecula, CA, USA), mAb-prohibitin (Abcam EP2803Y, Cambridge, UK) and nuclear lamin A/C (Abcam EPR4100), pAb-PDH (Thermofischer 18068-1-AP, Waltham, MA, USA), mAb-BGLF4 (mAb-BGLF4.01).

### 4.5. Coimmunoprecipitation (CoIP) and In Vitro Kinase Assay (IVKA)

Coimmunoprecipitation (CoIP) and in vitro kinase assay was performed according to [39,43], using the antibodies mAb-PDH (Abcam ab110334), mAb-mCherry (ab125096), mAb-Flag (Sigma F3165, St. Louis, MO, USA), mAb-TPE (thiophosphate-ester, Abcam ab92570), and Protein G-Dynabeads (25 µL, 10002D, Thermo Fisher Scientific, USA).

### 4.6. EBV-Specific qPCR-Based Antiviral Replication Assay and Determination of Cell Viability

The antiviral activity of drugs against EBV was measured by qPCR. The EBV-positive immortalized B cell line P3HR-1 was stimulated with 40 ng/mL TPA (12-O-tetradecanoyl-phorbol-13-acetate) to induce the lytic viral cycle. P3HR-1 cells were seeded in 96-well plates, simultaneously induced with TPA, and treated with antiviral compounds for 10 d, before cell culture medium samples were subjected to quantitative polymerase chain-reaction (qPCR) to assess the EBV lytic productivity. Thus, at 10 d post-treatment, samples were exposed to proteinase K digestion, followed by the EBV/BGLF5-specific qPCR analysis, using primers 5′-TGA CCT CTT GCA TGG CCT CT-3′ and 5′-CCT CTT TTC CAA GTC AGA ATT TGA C-3′; as well as, FAM probe 5′-CCA TCT ACC CAT CCT ACA CTG CGC TTT ACA-3′ [36,40]. For the determination of cell viability, the resazurin-based assay was performed in a 96-well plate format as described in [70].

### 4.7. Quantitative Sox Peptide-Based In Vitro Kinase Assay (qSox-IVKA)

To assess the specific BGLF4 inhibitory activity of the compounds, a qSox-IVKA was performed according to [43]. A full-length recombinant product (amino acids 1-429) of BGLF4 was produced and purified, using the commercial Leishmania Lexsy expression system (Jena Bioscience GmbH, Jena, Germany), to obtain a stock solution of 0.1 mg/mL, which proved to be stable upon storage at −80 °C. The product comprised kinase activity, as monitored by qSox-IVKA, and was thus used to equilibrate assay conditions (Figure S4), while the actual BGLF4–PDH relevant measurements were then performed with transient transfection-derived proteins (BGLF4-Flag; Figure 5). The kinase-specific sensor peptide AQT0258 (AssayQuant Inc., Marlborough, MA, USA) was used for this assay. The indicated compounds, including the DMSO control, were added at 10-fold concentration and measured in triplicates with 50 µL per reaction.

### 4.8. Indirect Immunofluorescence Assay and Confocal Laser-Scanning Microscopy

For immunofluorescence detection, sample numbers of 1.5 × 10^6^ TPA-treated (80 ng/mL, 4 d) P3HR-1 cells were seeded onto 0.05 mg/mL collagen-coated coverslips (Pan Biotech, Aidenbach, Germany, P06-20030) in serum-free medium to promote cell attachment. Cells were fixed on the same day with 10% formalin solution for 10 min at room temperature (Sigma Aldrich, St. Louis, MO, USA). Subsequently, cells were permeabilized with 0.2% Triton X-100 solution for 20 min at 4 °C (Roth, Karlsruhe, Germany). Another incubation with 2 mg/mL human γ-globulin Cohn fraction II (Merck, Darmstadt, Germany; 30 min, 37 °C) was performed to block nonspecific staining. Indirect immunofluorescence staining was carried out by incubating cells with primary antibody (pAb-PDH, Thermofischer 18068-1-AP; mAb-BGLF4.01, own repository; pAb-MDH2, Invitrogen) diluted in PBS for 60 min at 37 °C. After washing, a following incubation with diluted dye-conjugated secondary antibody (30 min, 37 °C) was performed. After additional washing steps, cells were mounted using VECTASHIELD^®^ Mounting Medium with DAPI (Vector Laboratories, Burlingame, CA, USA), and glass coverslips were sealed with nail polish. Confocal laser scanning microscopy was performed on a TCS SP5 microscope equipped with a 63× HCX PL APO CS oil immersion objective (Leica Microsystems, Mannheim, Germany). Image processing was conducted with LAS AF software (version 2.6.0, build 7266; Leica Microsystems, Mannheim, Germany) and further edited using Photoshop CS5 (Adobe, San José, CA, USA).

### 4.9. Determination of Intracellular Pyruvate Dehydrogenase (PDH) Activity

To determine the levels PDH activity in EBV-positive cells, two commercially available assay kits were applied according to the instructions of the manufacturers (all measurements in technical triplicates). For the pyruvate dehydrogenase (PDH) Enzyme Activity Microplate Assay Kit (ab109902, Abcam, Waltham, MA, USA), P3HR-1 cell cultures of 60 mL per sample were grown under normal nonstimulated or TPA-stimulated conditions as described in Section 4.6. After 4 d of treatment, cells were pelleted and washed. Cell pellets were lysed in volumes of 100 µL to determine total protein contents using standard Pierce measurement. Normalized samples of 142 µg each per PDH assay score were then subjected to precoated strips, provided with the kit, and subjected to photometric signal determination at 450 nm. For the Pyruvate Dehydrogenase Activity Assay Kit (MAK567, Sigma Aldrich, St. Louis, MO, USA), P3HR-1 cell cultures of 2 mL per sample were grown under the conditions described above. Total cell contents were used for photometric signal determination at 450 nm.

## Figures and Tables

**Table 1:** Mass spectrometry analysis based on CoIP with mAb-BGLF4.01 using total lysates of IgG-induced Akata-BX1 cells *.

Gene	Entry	Protein	WSC BGLF4/Beads Ctrl (Quotient)		WSC ≥ 10 (Absolute Values)	
			Analysis 1	Analysis 2	Analysis 1	Analysis 2
*DLAT*	A0A024R3D8_HUMAN	Acetyltransferase component of pyruvate dehydrogenase complex	168	11	321	122
*PDHA1*	ODPA_HUMAN	Dehydrogenase E1 component subunit alpha, somatic form, mitochondrial	224	6	224	35
*PDHB*	ODPB_HUMAN	Dehydrogenase E1 component subunit beta, mitochondrial	103	6	205	63
*PDHX*	ODPX_HUMAN	Pyruvate dehydrogenase protein X component, mitochondrial	64	7	64	51
*DLD*	DLDH_HUMAN	Dihydrolipoyl dehydrogenase, mitochondrial	(36) ^#^	28	36	56
*NCL*	NUCL_HUMAN	Nucleolin	29	(14) ^#^	29	14
*RPLP0*	RLA0_HUMAN	Large ribosomal subunit prot. uL10	(21) ^#^	14	21	14
*RPS4X*	RS4X_HUMAN	Small ribosomal subunit protein	5	6	15	13
*RPL10A*	RL10A_HUMAN	Large ribosomal subunit protein	(10) ^#^	(1) ^#^	10	1
*BGLF4*	YP_001129482.1	BGLF4 (Epstein–Barr virus, AG876)	7	5	43	35

* Two independent replicates of mass spectrometry analysis were performed, and WSC scoring hits of both settings were presented as relative values (Analyses 1 and 2). Cellular pyruvate dehydrogenase (PDH) was noted as the BGLF4 interactor with highest WSCs (blue frame). ^#^ Absolute WSCs for beads control were 0/minimal (thus, absolute values are given, no quotient possible).

## Data Availability

The original contributions presented in this study are included in the article/Supplementary Material. Further inquiries can be directed to the corresponding author.

## Supplementary Materials

The following supporting information can be downloaded at: https://www.mdpi.com/article/10.3390/ijms27062627/s1.
