# Supplementary material for: Identification of a New Phosphorylated Host Interactor of the Epstein–Barr Virus (EBV) Kinase BGLF4 Suggests Key Points for EBV-Specific Antiviral Drug Targeting
Source: Int J Mol Sci. 2026 Mar 13;27(6):2627. doi: 10.3390/ijms27062627 (PMC13026206; doi:10.3390/ijms27062627)
Supplement: Supplementary file 1 [file ijms-27-02627-s001.zip › Supplementary Materials_Kögler et al.pdf]

# *Supplementary Materials*

# Identification of a new phosphorylated host interactor of the Epstein-Barr virus (EBV) kinase BGLF4 suggests key points for EBV-specific antiviral drug targeting

**Melanie Kögler [1], Christina Wangen [1], Alena Hammerschmitt [1], Debora Obergfäll [1], Friedrich Hahn [1,§] and Manfred Marschall [1,*]**

[1] Harald zur Hausen Institute of Virology, Friedrich-Alexander-Universität Erlangen-Nürnberg (FAU), Schlossgarten 4, 91054 Erlangen, Germany; melanie.koegler@fau.de, christina.wangen@uk-erlangen.de, alena.hammerschmitt@fau.de, debora.obergfaell@fau.de, manfred.marschall@fau.de

§ Present address: Institute of Virology, Ulm University Medical Center, Ulm, Germany; friedrich.hahn@uni-ulm.de.

* Correspondence: manfred.marschall@fau.de, Tel.: ++49 9131 8536096

---

**Figure: S1.** Complete set of mass spectrometry data referring to Table 1 (see Supplementary Figure S1_Kögler et al.xlsx).

---

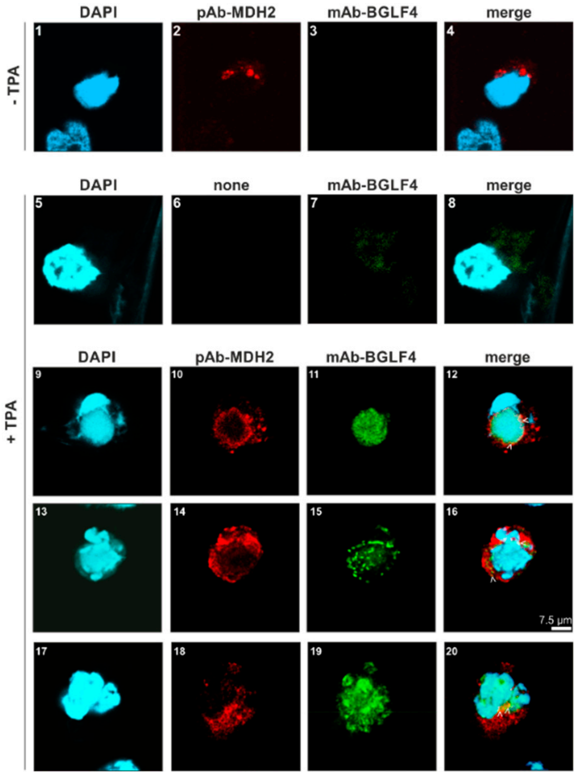


**Figure S2.** Confocal imaging of BGLF4 and malate dehydrogenase 2 (MDH2), used as a mitochondrial marker protein (refers to Figure 3). P3HR-1 cells were analyzed either stimulated with TPA (80 ng/mL for 4d, images 5-20) or remained unstimulated (images 1-4). Cells were cultured on collagen-coated coverslips (0.05 mg/mL) in serum-free medium and subjected to indirect immunofluorescence staining with antibodies specific for BGLF4 and MDH2. BGLF4 was visualized using Alexa Fluor 555 (red), whereas MDH2 was detected using Alexa Fluor 488 (green). Nuclear counterstaining was performed with DAPI. White arrows indicate regions of potential mitochondrial colocalization; scale bar, 7.5 µm.

---

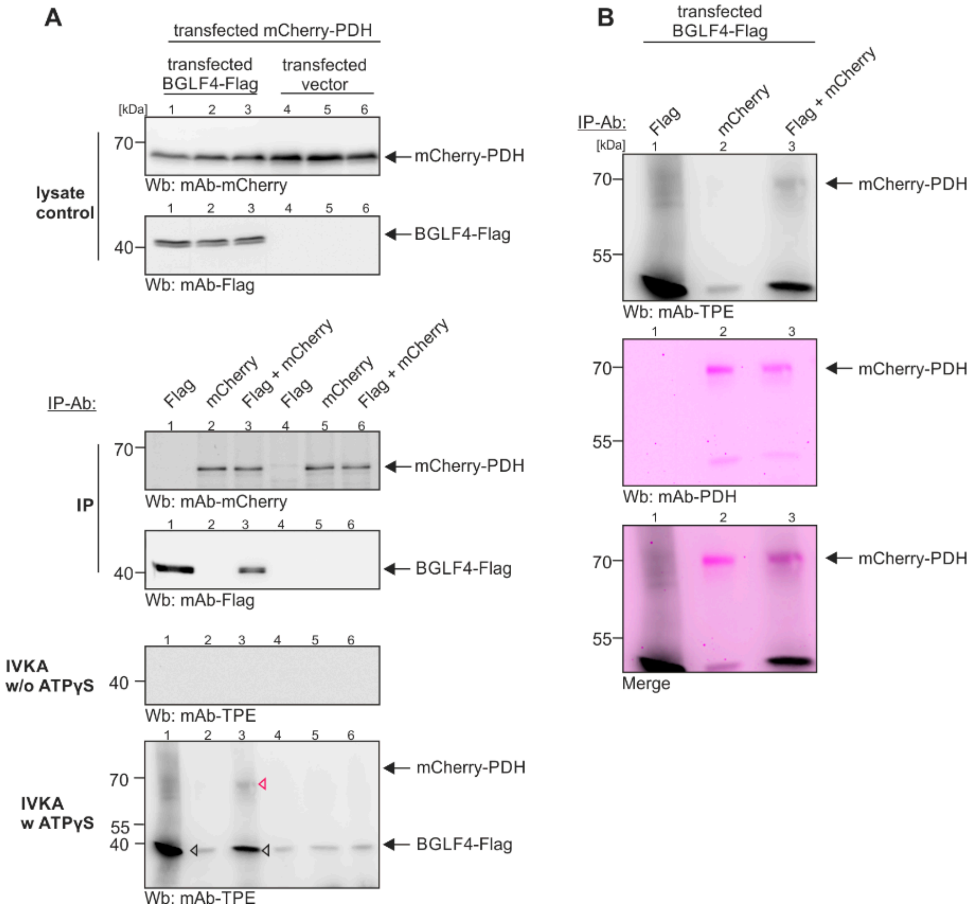


**Figure S3**. *In vitro* phosphorylation of PDH by BGLF4 using proteins derived from plasmid-cotransfected 293T cells (as a second experimental replicate referring to Figure 4). Cells were transiently transfected with expression plasmids encoding an autofluorescent mCherry-PDH protein, or BGLF4-Flag, or empty vector, as indicated. Two days post-transfection, aliquots of cell lysates (50 µL) were collected as input lysate controls (**A**), while the remaining lysates (450 µL) were subjected to immunoprecipitation using the antibodies indicated above the panels (IP-Ab). Immunoprecipitated proteins were subjected to *in vitro* kinase assay (IVKA) in the presence (w) or absence (w/o) of ATPγS. Distinct phosphorylation signals corresponding to BGLF4 autophosphorylation (approx. 40 kDa; black triangle) and mCherry-PDH (approx. 68 kDa; red triangle) were detected exclusively in reactions containing ATPγS, whereas no specific bands were observed in the absence of ATPγS, confirming assay specificity. (**B**) IVKA blots (lanes 1–3) were restained with a PDH-specific antibody (pink signal; inset panels) to confirm the PDH specificity of the BGLF4-mediated phosphorylation band.

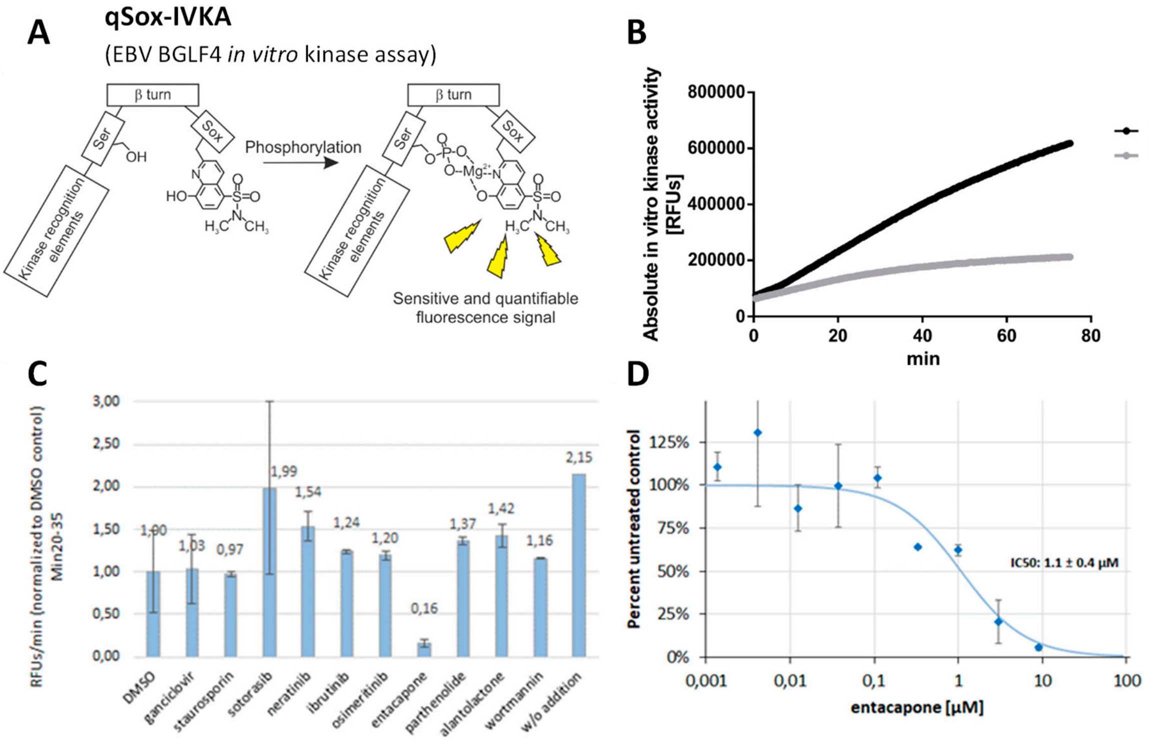


**Figure S4**. Establishment of a quantitative EBV-specific *in vitro* kinase assay (qSox-IVKA): screening for BGLF4-inhibitory small molecules. **(A-B)** EBV kinase BGLF4-specific qSox-IVKA: This newly established *in vitro* kinase assay was applied for the determination of EBV kinase BGLF4 by measuring the catalytic activity of transiently expressed BGFL4-Flag through the phosphorylation-mediated quantitation of a fluorescence-based Sox substrate peptides (i.e. phosphorylation-specific binding of the Sox peptide to a serine residue next to the kinase recognition element). Fluorometric determination of this BGLF4 activity was performed in a reaction kinetics over approx. 80 min (as to be background-corrected using vector control). **(C-D)** First experimental nomination of kinase inhibitors with anti-BGLF4 activity: A pilot screening with currently available, clinically relevant kinase and other inhibitors in the described qSox-IKVA system for measuring EBV kinase BGLF4 revealed a concentration-dependent curve of BGLF4 inhibition through a first in vitro hit compound entacapone (entacapone was diluted in reaction buffer to a serial concentration row of 9.0 to 0.0013 µM, and applied to the qSox-IVKA with 50 nM recombinant BGLF4, derived from the commercial Leishmania Lexsy expression system), to obtain a stock solution of 0.1 mg/ml, which proved to be stable upon storage at -80 °C. leishmania expression system ). The kinase activity was measured in a Victor-Multilabel-Reader all 30 sec for a duration of approx. 80 min; see that the steepness of signal increase per min was normalized against a DMSO solvent control; given are mean values ± SD as derived from two independent measurements both performed in duplicates.

---

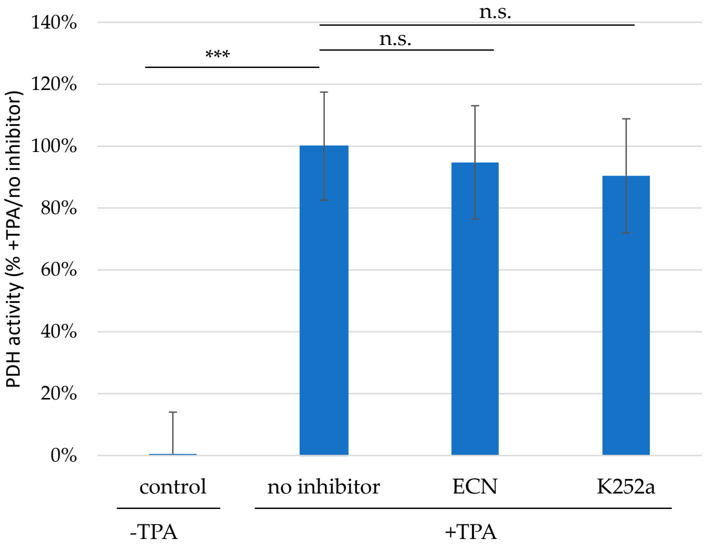


**Figure S5.** Measurement of PDH activity in TPA-stimulated EBV-positive Akata-BX1 cells, in the absence or presence of BGLF4-directed inhibitors (applying the PDH assay MAK567, as a second independent readout system referring to Figure 8). Akata-BX1 cells were cultivated in media volumes of 6 mL per sample for 4 d. PDH activity was determined according to instructions of the manufacturer described in section 4.9. Measurements were performed in technical triplicates. Mean values ± SD are shown, and Student's t-test was applied for statistical evaluation. ***, p> 0.001; n.s., not significant.
